# First report of *Centrocestus formosanus* infection associated with mass mortality in the critically endangered Chinese Bahaba (*Bahaba taipingensis*)

**DOI:** 10.1016/j.ijppaw.2026.101280

**Published:** 2026-09-01

**Authors:** Zhen Wu, Liwen Xu, Lin Yan, ChiHai Ji, Peiqi Tang, Juan Feng, Kuoqiu Yan, Zhixun Guo, Yiqin Deng

**Affiliations:** a Key Laboratory of South China Sea Fishery Resources Exploitation & Utilization Ministry of Agriculture and Rural Affairs, South China Sea Fisheries Research Institute, Chinese Academy of Fishery Sciences, Guangzhou, 510300, China; b Sanya Tropical Fisheries Research Institute, Sanya, Hainan, 572025, China; c Guangdong Bluegen Marine Biotechnology Co., Ltd, Huizhou, 516300, China; d Southern Marine Science and Engineering Guangdong Laboratory (Zhuhai), China; e College of Marine Sciences, South China Agricultural University, Guangzhou, 510642, China

**Keywords:** 28S rRNA gene, *Bahaba taipingensis*, *Centrocestus formosanus*, Gill histopathology, Parasite infection intensity

## Abstract

*Centrocestus formosanus* is a pathogenic heterophyid trematode traditionally known to infect freshwater teleosts, but increasingly recognized for its expanding range into brackish and coastal ecosystems.This study documents the first natural spillover of *C. formosanus* into the critically endangered marine euryhaline fish, Chinese bahaba (*Bahaba taipingensis*), which naturally inhabits both high-salinity seawater and brackish estuaries. A devastating disease outbreak accompanied by mass mortality occurred between November 2025 and January 2026 at an aquaculture base in Panyu, Guangzhou, China. *B. taipingensis* was maintained in low-salinity brackish water (3–5‰) as a result of its euryhaline tolerance. Notably, this low salinity (3–5‰) is known to be the most permissive range for cercarial survival and host-seeking behavior, thereby creating an ecological window for trematode infection. Among the 10 infected fish examined, all exhibited excessive gill mucus and dense macroscopic white spots across the gill filaments. Encysted metacercariae were morphologically identified and molecularly confirmed via 28S rRNA gene sequencing, displaying 99.96% sequence identity to reference strains *C. formosanus* (MG738251.1 and OR671446.1).Parasitological quantification revealed an exceptionally high mean infection intensity (3832 ± 473 parasites per fish). Histopathological evaluation demonstrated catastrophic branchial remodeling, prominently characterized by host-derived granuloma encapsulation, pronounced tissue fibrosis, lamellar epithelial hypertrophy, widespread lamellar atrophy and fusion, and severe localized swelling of the gill filaments. Diagnostic screening ruled out the presence of concomitant bacterial or viral pathogens. This first documentation of *C. formosanus* in this euryhaline sciaenid underscores the acute vulnerability and extremely low tolerance of *B. taipingensis* to this heterophyid fluke, highlighting a critical and previously unrecognized parasitic threat to the conservation and population recovery of this endangered species.

## Highlights

• λ*Centrocestus formosanus* infected *Bahaba taipingensis* is reported for the first time.
• λLow-salinity culture (3–5‰) provides a permissive window for infection.
• λExceptionally high mean infection intensity reached 3832 ± 473 PPF.
• λSevere destruction and remodeling of gills caused fatal hypoxic suffocation.
• λ*C. formosanus* poses a major biotic threat to endangered fish recovery.

## Graphical abstract

**Figure:**
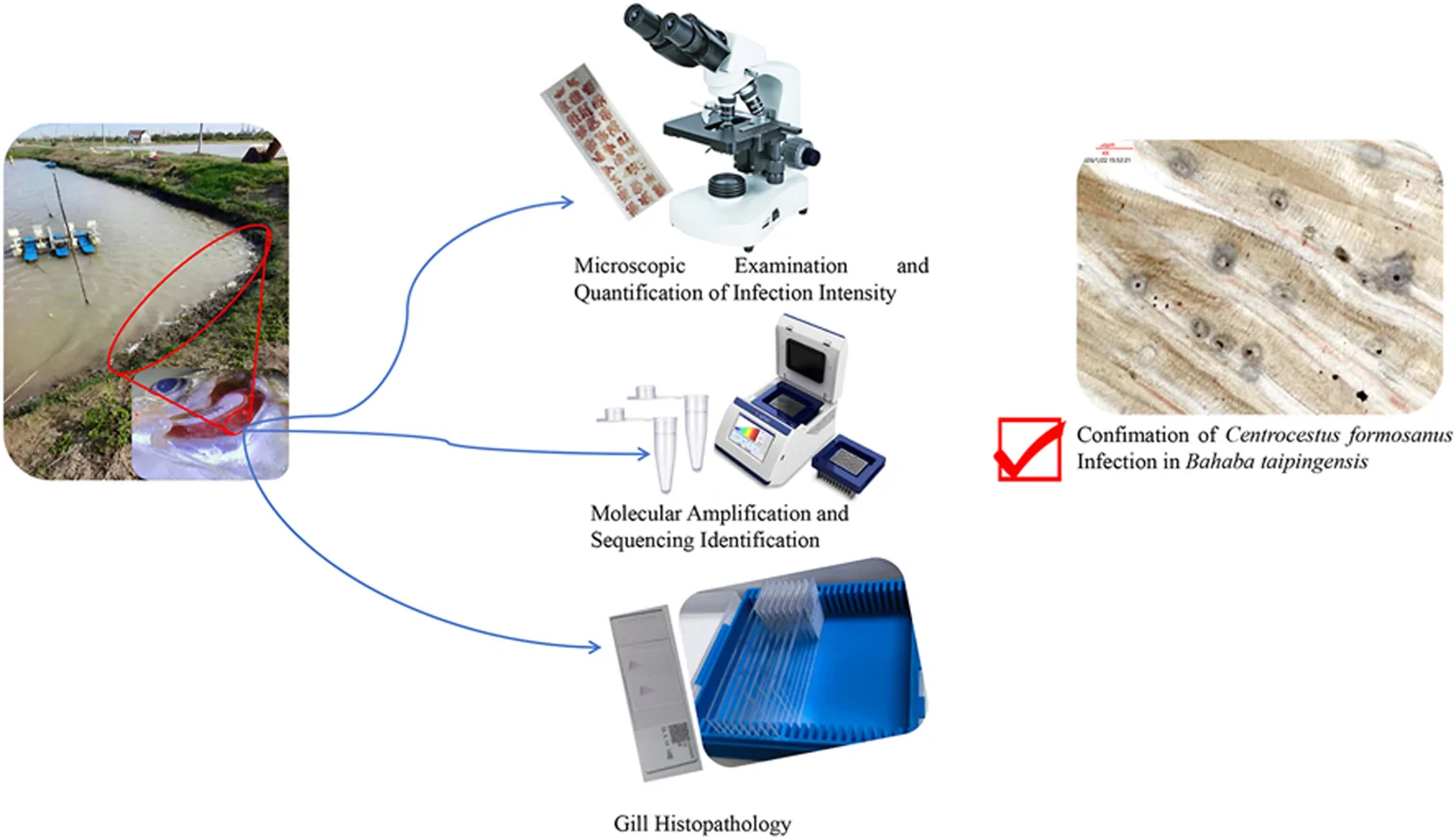

## 1 Introduction

The family Heterophyidae comprises a group of small intestinal trematodes that parasitize birds, mammals, and humans as definitive hosts, serving as major causative agents of foodborne zoonotic diseases globally (Chai and Jung, 2019). Among these, *Centrocestus formosanus* is a highly pathogenic species, typified morphologically by the presence of 30–36 circumoral spines arranged around the oral opening of the adult worm (Khallaf et al., 2025). The life cycle of *C. formosanus* is complex and multi-staged: embryonated eggs are shed into water bodies via the feces of definitive hosts and are subsequently ingested by the first intermediate snail host; within the snail's gut, the eggs hatch into miracidia, which then penetrate the intestinal wall to initiate intramolluscan development, primarily invasival snail *Melanoides tuberculata*. Following asexual intramolluscan development, cercariae are produced within rediae and subsequently emerge from the mollusc into the water, where they await passive inhalation by passing fish. Upon contact with fish gills, the cercariae attach and then penetrate the gill epithelium, encysting as metacercariae—typically encompassing various freshwater teleosts, including grass carp and common carp, though expanding historical boundaries to include euryhaline and brackish-water hosts such as tilapia (Jithila et al., 2022; Kamchoo and Chai, 2023). The life cycle is completed when piscivorous birds or mammals (including humans) ingest raw or undercooked fish harboring viable metacercariae, which excyst in the small intestine and mature into adult flukes (Mitchell et al., 2005). The encystment of metacercariae within fish gills frequently triggers acute to chronic inflammatory cascades, marked by extensive cartilaginous and epithelial hyperplasia. This structural remodeling causes severe gill damage, respiratory dysfunction, hypoxic distress, and extensive mortality in aquaculture systems (Khallaf et al., 2025).

*Centrocestus formosanus* exhibits a cosmopolitan distribution, with widespread records across wild and farmed fish populations in Asia and the Americas, including fountain darter (*Etheostoma fonticola*), greenhouse darter (*Etheostoma lepidum*), and yellow bullhead (*Ameiurus natalis*), as well as several aquaculture species such as channel catfish (*Ictalurus punctatus*) and golden shiner (*Notemigonus crysoleucas*), poses a critical threat to the survival of the fountain darter (*Etheostoma fonticola*) and several other federally endangered fish species in the United States (Mitchell et al., 2000; McDermott et al., 2014). In the United States, this exotic parasite, which is native to Asia, has inflicted substantial economic losses on the tropical ornamental fish industry since the early 1980s and poses a critical threat to the survival of the federally endangered fountain darter (*Etheostoma fonticola*) (Mitchell et al., 2000). In Brazil, epidemiological surveys of wild cichlids (*Australoheros facetus*) revealed a 100% prevalence, with a mean infection intensity of 134 PPF and a maximum burden reaching 2510 PPF (Pinto and de Melo, 2012). Similarly, the ornamental fish sector in Mexico has been heavily impacted, with a prevalence of 66.7% (20/30) among surveyed goldfish farms, where infections were strongly correlated with elevated mortality rates (Ortega et al., 2011).In China, extensive surveys have demonstrated high susceptibility among economically important freshwater fishes. In bighead carp (*Aristichthys nobilis*), the prevalence of metacercariae on gill filaments and gill rakers reached up to 100%, with mean values of 83.33% and 61.13%, respectively (Zeng and Liao, 2001). Concurrently, the infection rate in grass carp (*Ctenopharyngodon idella*) ranged from 2.1% to 100% across regions (Zeng and Liao, 2000). Collectively, these lines of evidence emphasize that *C. formosanus* possesses a remarkably broad host spectrum and formidable pathogenic potential, designating it as a high-priority pathogen requiring stringent biosecurity control.

No formal report has described *C. formosanus* infection in the Chinese bahaba (*B. taipingensis*). The *B. taipingensis* is a large, endemic marine sciaenid with immense economic and ecological conservation value. As a euryhaline marine teleost, it was historically widely distributed across the southeastern coast and major estuarine waters of China. Its wild populations have severely declined due to historical overfishing and coastal habitat degradation (Huang et al., 2020), and it is currently critically endangered and designated as a National First-Class Protected Wildlife in China (Liu, 2020). Consequently, artificial propagation and stock enhancement have become the primary lifelines for the recovery of this species (Mo et al., 2023). However, both in the wild and in captivity, disease problems are becoming increasingly prominent. Known pathogens affecting *B. taipingensis* include *Edwardsiella tarda* (Li et al., 2024), an unidentified pathogen causing white spot disease along with *Saprolegnia* spp. (Guo et al., 2013), and various ectoparasites such as *Caligus orientalis* (Guo and Cai, 2018) and unidentified branchiuran “fish lice” (Guo and Yang, 2018). Consequently, systematic disease investigations in *B. taipingensis* are urgently needed to establish precise prophylactic technologies and safeguard the restoration of this critically endangered resource.

In the present study, we identified *C. formosanus* as the primary etiological agent responsible for a severe, sustained mass mortality event in pond-cultured *B. taipingensis*. We present a comprehensive analysis of the clinical manifestations, absolute parasite burdens, histopathological alterations, and molecular phylogenetic characteristics of this novel infection case. These findings provide vital insights into the pathogenesis of heterophyid trematodes in brackish-water aquaculture settings and offer a scientific basis for the timely implementation of targeted disease management protocols in endangered sciaenid aquaculture.

## 2 Materials and methods

### 2.1 Clinical background and sample collection

**Fig. 1:**
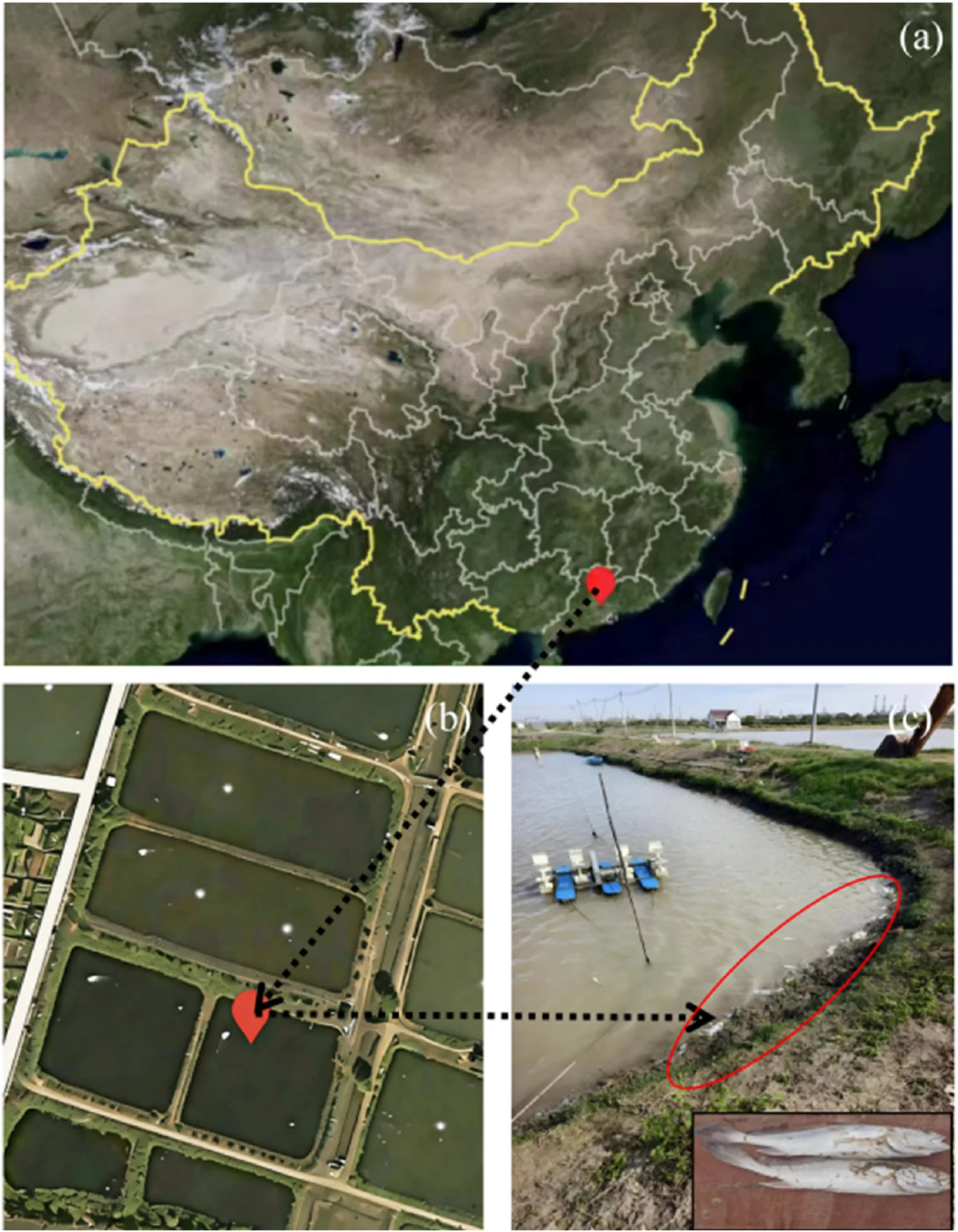
Geographic location and features of the sampling site for diseased *Bahaba taipingensis*. (a) Satellite map showing the geographic coordinates (E113°30′49″, N22°59′56″) of the sampling site in Panyu District, Guangzhou City, Guangdong Province, China; (b) Enlarged satellite imagery of the aquaculture base where the host fish were cultured; (c) Overview of the aquaculture ponds at the base; the red circle delineates dead and moribund fish floating on the water surface.

Between November 18, 2025, and January 28, 2026, a severe and sustained mortality event occurred among cultured juvenile *B. taipingensis* (average total length: ∼25 cm; average body weight: ∼130 g) maintained at the Panyu aquaculture base, Guangzhou, China (E113°30′49″, N22°59′56″) of Guangdong Bluegen Marine Biotechnology Co., Ltd., Huizhou, China (licence for domestication and breeding of endangered aquatic species of PRC #2026YL0083) (Fig. 1a and b). While the species typically thrives under full-strength marine conditions, such as the 20-35‰ salinity regime maintained at the corporate headquarters in Huizhou, its intrinsic euryhalinity allows for sustained aquaculture within estuarine settings. At the Panyu aquaculture base, fish were routinely maintained in low-salinity brackish water (3–5‰), which is the standard salinity used at this facility.During the peak of the outbreak, daily mortality escalated to hundreds of individuals per pond, with moribund and dead fish floating on the water surface (Fig. 1c). Affected fish exhibited no distinctive macroscopic lesions on the external body surface. For etiological diagnosis, 10 freshly dead fish were randomly sampled from the peak mortality period and immediately transported under refrigeration to the laboratory for comprehensive diagnostic analysis.

### 2.2 Clinical examination and sample collection

Post-mortem examination commenced with external somatic evaluation. Individual fish were weighed and measured for total length, and documented photographically. To evaluate parasitic infestation, segments of gill filaments were excised under sterile conditions, immersed in a Petri dish containing sterile physiological saline (0.9% NaCl), mounted as wet mounts on glass slides, and immediately examined via light microscopy. Subsequently, a longitudinal incision was made along the ventral midline to expose the visceral cavity for gross pathological evaluation.

Under strict aseptic conditions, the surfaces of the spleen and kidney were seared and incised. Tissue fluids were sampled using sterile inoculation loops and streaked onto Brain Heart Infusion (BHI) agar plates for bacterial isolation. Concurrently, representative tissue biopsies (∼50 mg) from the gills, spleen, kidney, and brain were harvested, placed into sterile 2 mL microcentrifuge tubes, and immediately flash-frozen in liquid nitrogen for subsequent nucleic acid extraction and molecular diagnostic assays. For histopathology, parallel tissue samples from the gills, spleen, kidney, and heart were immediately immersed in 4% buffered paraformaldehyde fixative and allowed to fix at room temperature for at least 24 h.

### 2.3 Quantification of parasite infection intensity

The 10 collected fish (designated Nos. 1–10) were utilized to assess absolute parasite burdens. Following somatic weight registration, all gill arches from both sides of each fish were systematically excised. The holobranchs were separated into individual hemibranchs, placed on glass slides, moistened with physiological saline, and gently compressed under a coverslip to optimize tissue transparency and flattening. Each lamella was systematically scanned under a light microscope to record all encysted metacercariae across all fields of view. The cumulative count from all gill arches was calculated to determine the total parasite burden per fish (Parasites Per Fish, PPF).

### 2.4 Nucleic acid extraction and cDNA synthesis

Frozen tissue samples from the gills, spleen, kidney, and brain of all 10 specimen fish were subjected to cryogenic grinding for 10 min using a automated tissue homogenizer to ensure complete cell lysis. Total nucleic acids (simultaneous DNA and RNA co-extraction) were extracted using the ALFA-SEQ Magnetic Aquatic Animal Pathogens DNA/RNA Kit I (FINDROP, NC303-03) according to the manufacturer's instructions. Total RNA from the visceral tissues was immediately reverse-transcribed into complementary DNA (cDNA) using the Evo M-MLV Reverse Transcription Kit (AGBIO, AG11728). The resulting genomic DNA and cDNA templates derived from internal organs were reserved for high-throughput qPCR pathogen screening, whereas genomic DNA extracted from gill tissues was utilized for 28s rRNA PCR amplification to identify the parasite.

### 2.5 Diagnostic screening via real-time quantitative PCR (qPCR)

To rule out concurrent infections, the tissue extracts were screened for common marine fish pathogens endemic to South China coastal aquaculture, including: *Vibrio harveyi* (VH); Nervous Necrosis Virus (NNV); Red Sea Bream Iridovirus (RSIV); Carpione rhabdovirus (CAPRV); *Cryptocaryon irritans* (CI); *Streptococcus iniae* (SI); *Nocardia seriolae* (NS); *Mycobacterium marinum* (MM); Real-time quantitative PCR (qPCR) assays were executed in a final reaction volume of 20 μL, comprising 10 μL of 2× Probe qPCR Mix, 0.8 μL each of forward and reverse primers (10 μM), 0.4 μL of the specific fluorogenic probe (10 μM), 2 μL of template nucleic acid, and nuclease-free ddH_2_O to volume. The assays were conducted on a real-time PCR platform using validated thermal profiles. Samples were verified as positive if they exhibited a characteristic sigmoidal amplification curve with a Cycle Threshold (Ct) value ≤ 35. Samples yielding no detectable fluorescence signal or Ct > 35 without true geometric amplification were categorized as negative. The diagnostic primer and probe sets utilized are summarized in Table 1.

**Table 1:** A total of eight primer-probe sets were utilized for real-time quantitative PCR (qPCR) diagnostic screening of eight common marine fish pathogens in *B*. *taipingensis.*

Primer/Probe Name	Sequence
VH-F	GCTGTTGTTGCTGTTGTTG
VH-R	CGTTGTTGTTGTTGTTGTTG
VH-Probe	FAM-CTGCTGCTGCTGCTGCTGCTG-BHQ1
NNV-F	CAACTGACARCGAHCACAC
NNV-R	CCCACCAYTTGGCVAC
NNV-Probe	FAM-TYCARGCRACTCGTGGTGCVG-BHQ1
RSIV-F	TGACCAGCGAGTTCCTTGACTT
RSIV-R	CATAGTCTGACCGTTGGTGATACC
RSIV-Probe	FAM-AACGCCTGCATGATGCCTGGC-TAMRA
CAPRV-F	CCAGAACCTGTTTGCGCTTG
CAPRV -R	AGTCCCTATTCACCACCCAGA
CAPRV -Probe	FAM-CTCACAGATAATGTGGCGGCAAAGAGGGAG-BHQ1
CI-F	TGACCAGCGAGTTCCTTGACTT
CI-R	CATAGTCTGACCGTTGGTGATACC
CI-Probe	FAM-AACGCCTGCATGATGCCTGGC-TAMRA
SI-tq-F	AGAAGAGATGAAAGCACAGATG
SI-tq-R	GTGTAGCCGTCTAAACCAATCA
SI-tqMan	FAM-CGCACGGTCACTAACGACAATGGAT-BHQ1
NS-tqF	CTGCTCAAGAAGGACCTG
NS-tqR	GTCCTTGTCCTTGGTGTA
NS-tqMan	FAM-ACTACGAGACCGACATCAAGAAGC-BHQ1
MM-tqF	GACAGCACAAGCCGAGGACG
MM-tqR	ACGATTCACAGTAAACGCAC
MM-tqMan	FAM-ACAGGCCGTTCGCTCAGTCGT-BHQ1

**Note:** VH, *Vibrio harveyi*; NNV, Nervous necrosis virus; RSIV, Red sea bream iridovirus; CAPRV, Carpione rhabdovirus; CI, *Cryptocaryon irritans*; SI, *Streptococcus iniae*; NS, *Nocardia seriolae*; MM, *Mycobacterium marinum*. FAM, 6-carboxyfluorescein; BHQ1, Black Hole Quencher 1; TAMRA, tetramethylrhodamine.

### 2.6 PCR amplification, sequencing, and phylogenetic analysis of the parasite

Following microscopic confirmation of heavy heterophyid trematode infestation in the gills, a diagnostic PCR assay targeting the 28S rRNA gene region for phylogenetic analysis of the Heterophyidae family was developed. The specific primer set designed comprised forward primer He-F (5′-GTTACTCTCCTTGGTCCGTGT-3′) and reverse primer He-R (5′-TTGGTCATTAGGCAATGTGGTG-3′). PCR amplification was conducted in a 50 μL reaction mixture containing 25 μL of 2× Accurate Taq Master Mix, 4 μL of the primer mixture (10 μM each), 1 μL of template DNA from gill tissue,and sterile ddH_2_O added to reach the final volume. The thermal cycling profile consisted of an initial denaturation at 94°C for 30 s; followed by 35 cycles of denaturation at 98°C for 10 s, annealing at 57°C for 30 s, and extension at 72°C for 1 min; with a final extension step at 72°C for 2 min. Amplification products were resolved via electrophoresis on a 1.5% agarose gel, and the target amplicons were excised and purified using a gel extraction kit. The purified DNA was subjected to bidirectional Sanger sequencing. The resulting consensus sequence (assembled using DNASTAR Lasergene SeqMan Pro) was evaluated via the online Basic Local Alignment Search Tool (BLASTn) against the NCBI GenBank database. Based on sequence homology, 23 representative reference sequences from related heterophyids were retrieved, and a phylogenetic tree was constructed via the Neighbor-joining (NJ) method under the p-distance model with 1000 bootstrap replicates using MEGA 12 software.

### 2.7 *In Vitro* cell culture and infectivity assays

To definitively screen for potential viral pathogens not covered by qPCR, tissue homogenates (10% w/v in PBS, pH 7.4) of the spleen, kidney, and brain from diseased fish were prepared. The homogenates were clarified via centrifugation at 10,000 g for 10 min at 4°C, and the resulting supernatants were passed through a 0.22 μm polyethersulfone sterile syringe filter. Monolayers of Fathead Minnow (FHM) cells cultured in Leibovitz's L-15 medium supplemented with 10% fetal bovine serum were inoculated with the sterile filtrates. Cells inoculated with sterile PBS served as negative controls. Following an adsorption period of 2 h at 28°C, the inoculum was replaced with maintenance medium containing 2% FBS. The cell monolayers were monitored daily for cytopathic effects (CPE) for a total duration of 7 days (168 h) post-inoculation.

### 2.8 Histopathology

Spleen, kidney, and gill tissue samples that had been fixed in paraformaldehyde fixative at room temperature for 24 h (as described in Section 2.2) were subjected to routine histopathological processing. This procedure included dehydration, clearing, paraffin embedding, and sectioning into 4-μm-thick sections. The sections were subsequently stained with hematoxylin and eosin (H&E) using standard techniques for histopathological evaluation. All sections were examined under a light microscope (Leica DFC495, Ernst Leitz, Wetzlar, Germany).

## 3 Results

### 3.1 Etiological determination and parasite quantification

**Fig. 2:**
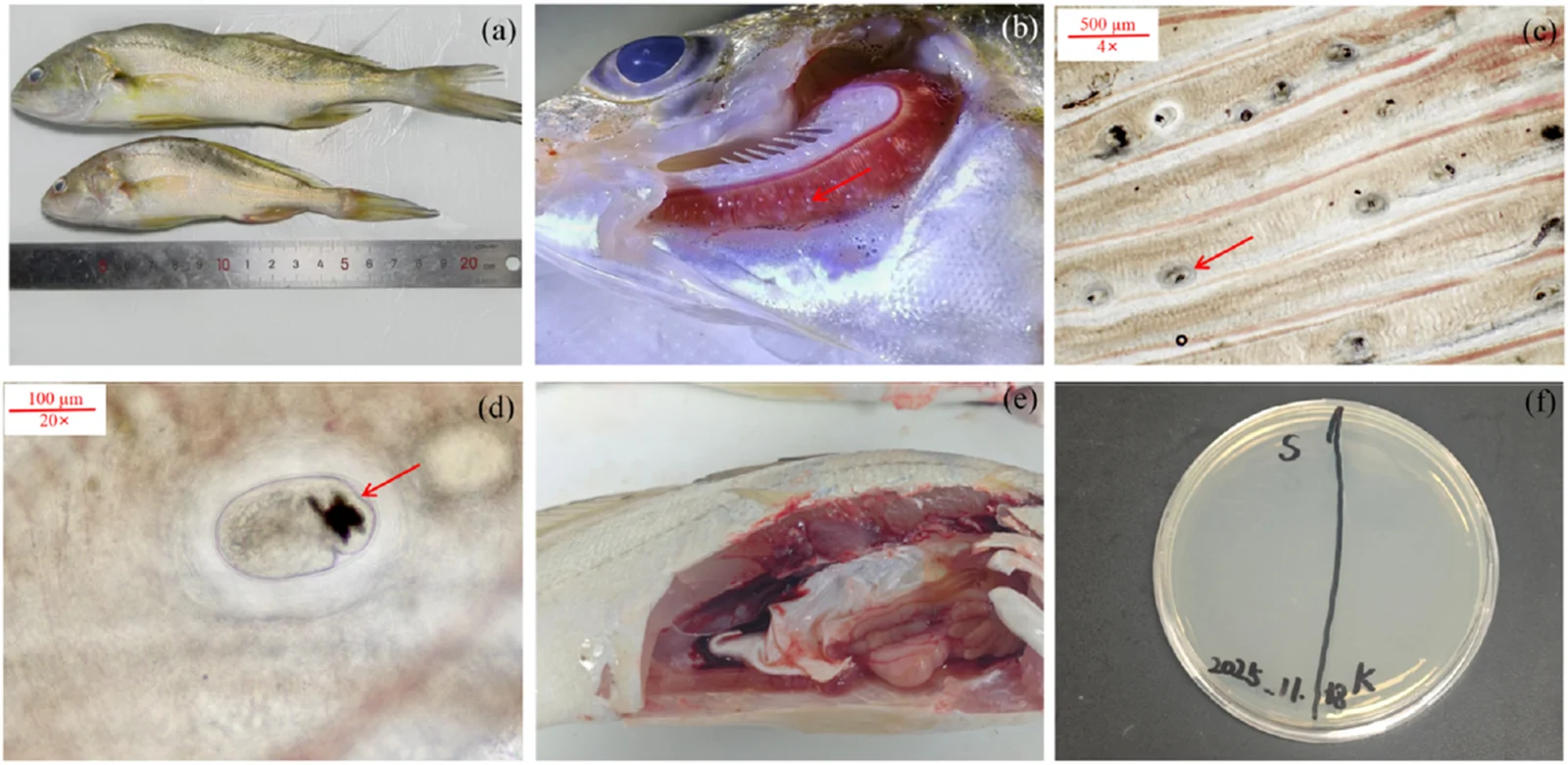
Clinical examination and pathological findings of diseased *B. taipingensis*. (a) Gross morphology of a moribund fish prior to necropsy, exhibiting an externally normal appearance; (b) *In situ* appearance of the gill filaments reveal numerous macroscopic white spots (encysted metacercariae) densely distributed along the lamellae (arrows); (c) Wet mount of branchial tissue under light microscopy, illustrating abundant encysted metacercariae (arrows); (d) The enlarged encysted metacercariae (arrows); (e) Exposed abdominal cavity demonstrating the absence of significant gross pathological alterations in the visceral organs; (f) Bacteriological isolation from internal organs on Brain Heart Infusion (BHI) agar plates after 16 h of incubation at 28°C, and no dominant bacterial colonies are visible in either the spleen (S) or kidney (K) zones.

**Fig. 3:**
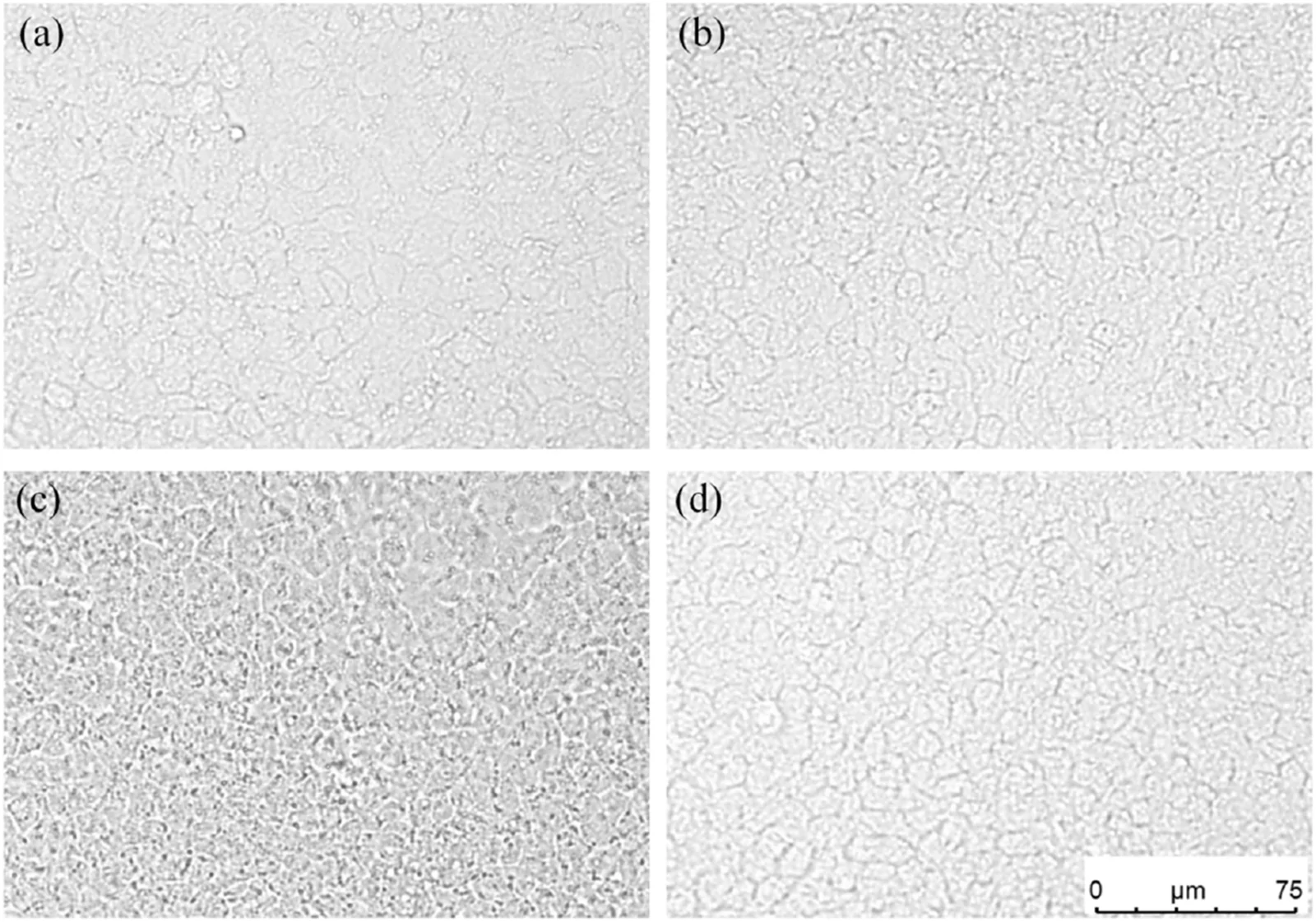
Evaluation of cytopathic effects (CPE) in Fathead Minnow (FHM) cells inoculated with tissue homogenates from diseased *Bahaba taipingensis* at 7 days post-infection (scale bar = 75 μm, 20× objective). Normal cellular morphology and an absolute absence of CPE are observed across all groups: (a) Negative control (PBS-inoculated); (b) spleen homogenate-inoculated; (c) kidney homogenate-inoculated; and (d) brain homogenate-inoculated cells. Micrographs (a) to (d) share the identical scale bar indicated in panel (d).

Gross clinical examination of freshly dead *B. taipingensis* revealed no conspicuous lesions, ulcerations, or hemorrhages on the external body surface (Fig. 2a). However, the gill filaments were densely studded with thousands of minute, opaque white spots (Fig. 2b). Direct wet-mount light microscopy confirmed that these white spots were thick-walled, oval encysted metacercariae embedded within the branchial tissue (Fig. 2c and d). Quantification of the parasitic load across the 10 sampled individuals revealed a mean absolute infection intensity of 3832 ± 473 PPF (range: 2262–7623 parasites per fish), demonstrating an extremely severe infection state. Internal anatomical evaluation revealed no gross lesions, organomegaly, or ascites within the peritoneal cavity (Fig. 2e). Microbiological cultivation on BHI plates remained completely devoid of dominant bacterial colonies after 16 h of incubation at 28°C (Fig. 2f). Furthermore, the cell culture infectivity assays showed no evidence of CPE in FHM cells inoculated with visceral tissue filtrates across the 7-day monitoring period, remaining morphologically indistinguishable from the PBS negative controls (Fig. 3). Concurrently, diagnostic screening via qPCR for all eight major endemic marine pathogens consistently yielded negative results across all tested organs (Table S1). Consequently, heavy branchial infestation by the trematode parasite was determined to be the exclusive primary etiological cause of the mass mortality event.

### 3.2 Molecular identification and phylogenetic analysis

Bidirectional sequencing of the 28S rRNA gene amplicon yielded a high-quality consensus sequence, designated as Pybt20251118. Comprehensive BLASTn analysis revealed that the sequence shares an exceptionally high genetic identity (99.96%) with validated GenBank reference sequences of *C. formosanus* (Accession Nos. MG738251.1 and OR671446.1), with a query coverage of 100%.

**Fig. 4:**
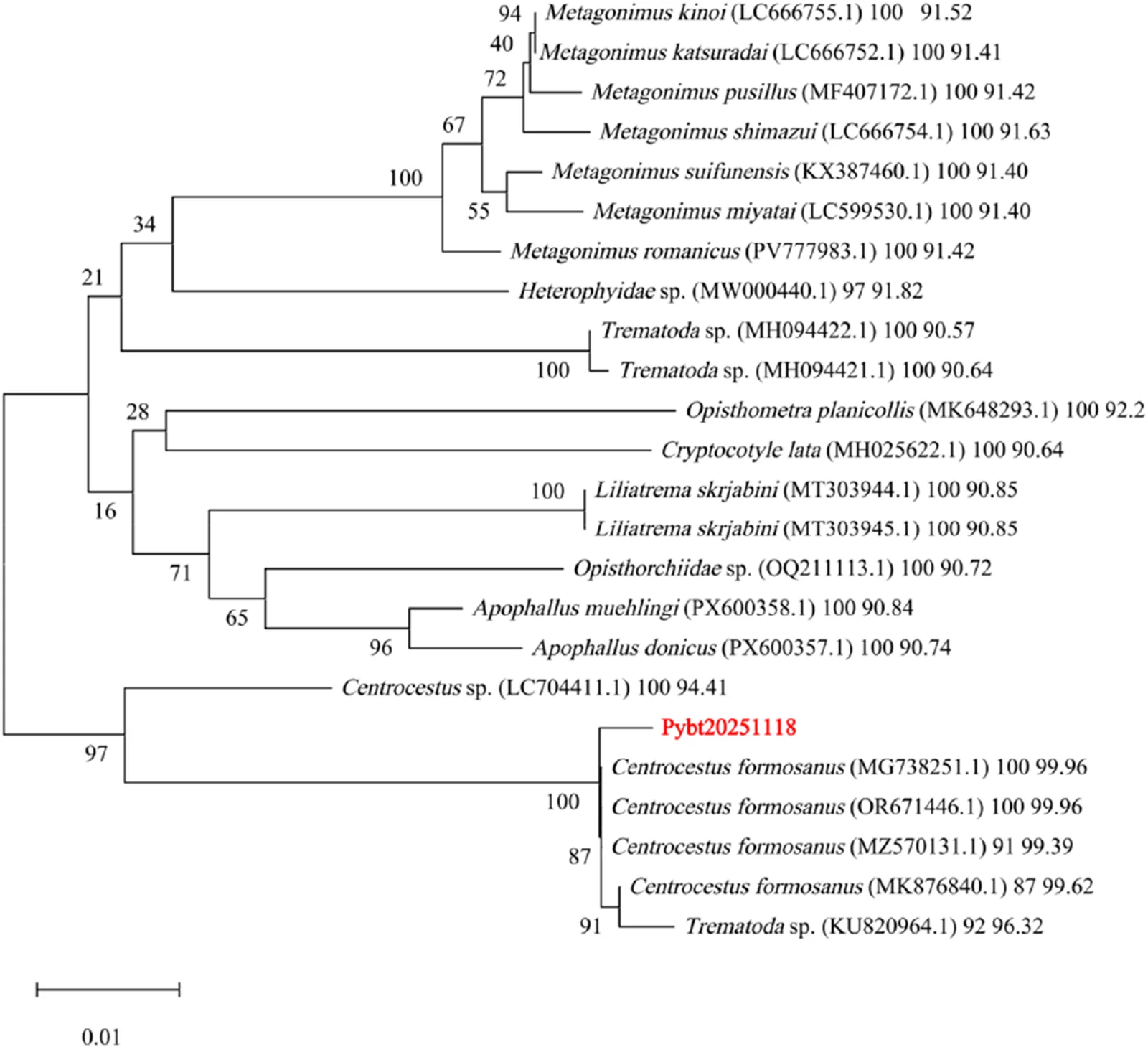
Neighbor-joining (NJ) phylogenetic tree of *Centrocestus formosanus* and related heterophyid trematode species derived from 28S rRNA gene sequences. The tree was reconstructed under the *p*-distance model using MEGA 12 software. Bootstrap values based on 1000 replicates are displayed at the corresponding nodes. Each operational taxonomic unit (OTU) label sequentially details the species name, GenBank accession number (in parentheses), query coverage (%), and percent sequence identity (%). The target consensus sequence obtained in the present study (Pybt20251118) is highlighted in bold red text. The scale bar represents an evolutionary distance of 0.01 substitutions per nucleotide site.

To establish its precise taxonomic position within the family Heterophyidae, a Neighbor-joining phylogenetic tree was reconstructed (Fig. 4). The topology shows that the investigated sequence Pybt20251118 clusters within the monophyletic *C. formosanus* clade with a maximum bootstrap support value of 100%, remaining distinct from other closely related heterophyid taxa such as *Metagonimus* spp., *Liliatrema* spp., and *Apophallus* spp. This high bootstrap consensus and minimal evolutionary distance firmly verified the parasite as *C. formosanus.*

### 3.3 Histopathology

**Fig. 5:**
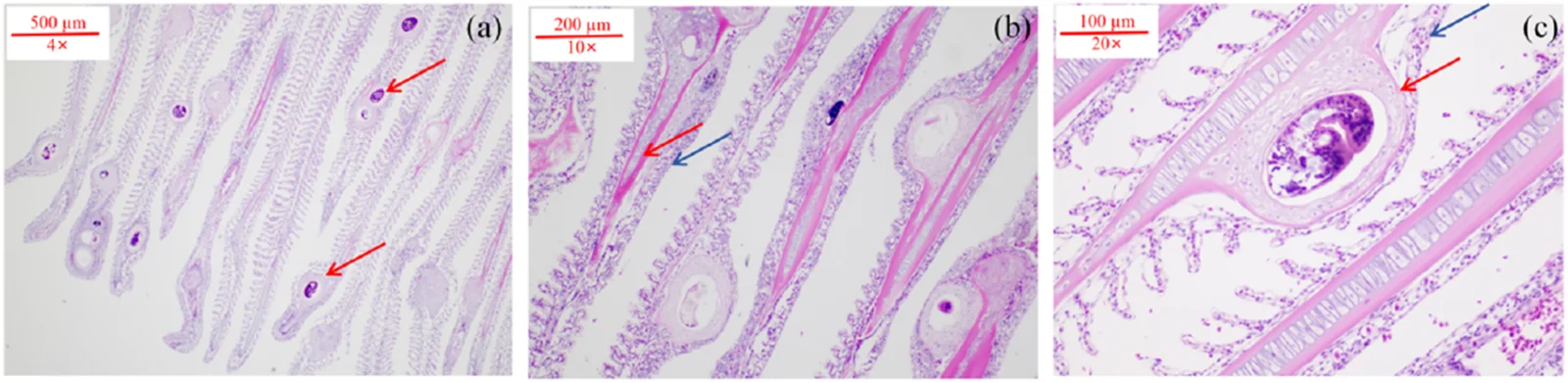
Histopathological observation of *B*. *taipingensis* gill tissues infected with *C*. *formosanus* metacercariae (H&E staining). (a) A large number of *C. formosanus* metacercariae (red arrows) are observed parasitizing the gill filaments of *B. taipingensis*, causing prominent localized swelling and deformation of the infected filaments (4× objective, scale bar = 500 μm); (b) Various pathological changes induced by the parasitism of metacercariae in the gill filaments are demonstrated, including distinct fibrosis (red arrows) and inflammatory response (blue arrows) in tissues surrounding the cartilaginous support (10× objective, scale bar = 200 μm); (c) A single metacercaria encysted within the gill filament, the metacercaria is encapsulated by a granuloma (red arrows) formed by host connective tissue proliferation, accompanied by the atrophy and fusion of surrounding gill lamellae (blue arrows) (20× objective, scale bar = 100 μm).

Histopathological examination revealed that the infection of *C. formosanus* metacercariae caused severe structural damage and prominent host immune responses in the gill tissues of *B. taipingensis*. At low magnification, a substantial number of metacercariae were densely aggregated within the gill filaments and the bases of the gill lamellae, resulting in severe localized swelling, thickening, and morphological deformation of the affected filaments (Fig. 5a). More characteristic pathological lesions were identified under medium and high magnifications: due to the mechanical compression and chronic irritation from the parasites, distinct fibrosis occurred in the tissues surrounding the cartilaginous support, accompanied by explicit inflammatory cell infiltration (Fig. 5b). Around individual metacercariae, typical granulomatous structures derived from host connective tissue proliferation were observed tightly encapsulating the parasites; moreover, owing to the mechanical displacement by the metacercarial nodules, the adjacent normal gill lamellae exhibited pronounced compressive atrophy, degeneration, and fusion (Fig. 5c). The extensive destruction and remodeling of the gill filaments and lamellae strongly indicate that this parasitic infection severely compromises the gas exchange and respiratory physiological functions of *B. taipingensis*.

In stark contrast to the gills, histopathological examination of the visceral organs revealed no structural abnormalities. The liver architecture displayed normal hepatic cord arrays with distinct sinusoids, and lacked signs of vacuolar degeneration, focal necrosis, or inflammatory cell aggregates (Fig. S1a and S1b). The spleen displayed a well-differentiated red and white pulp distribution, with no evidence of macrophage aggregate proliferation or necrotic foci (Fig. S1c). The myocardium showed regular, uncompromised interlacing myofibrillar arrangements devoid of interstitial edema or leukocyte infiltration (Fig. S1d). These systemic findings confirmed that the pathological damage was strictly localized to the respiratory apparatus.

## 4 Discussion

In this study, we conducted a systematic investigation into a devastating, high-mortality outbreak affecting critically endangered Chinese bahaba (*B. taipingensis*) at juveniles a aquaculture base in Panyu, Guangzhou, China. Utilizing clinical necropsy, histopathological profiling, and molecular sequencing, we conclusively established that *C. formosanus* was the causative agent of this mass mortality event. The high absolute parasite burden (3832 ± 473 PPF) coupled with severe branchial structural disruption, in the total absence of concomitant viral or bacterial pathogens, provides definitive evidence that this heterophyid trematode is highly pathogenic to *B. taipingensis*. As a marine euryhaline fish, *B. taipingensis* naturally tolerates wide salinity variations. This osmotic plasticity enables long-term aquaculture under estuarine conditions, such as the 3-5‰ brackish water environment at the Panyu facility, distinct from the 20-35‰ seawater system at the Huizhou headquarters. Critically, this 3-5‰ range coincides with the permissive osmotic threshold for the survival of the invasive snail *M. tuberculata* and the shedding of *C. formosanus* cercariae (Jithila et al., 2022; Kamchoo and Chai, 2023). The host's broad salinity tolerance thus created an artificial ecological crossroads, facilitating a freshwater parasite spillover into a marine-derived teleost entirely naive to freshwater pathogens. This study fills a critical host record gap for *C. formosanus* in a unique, endemic marine teleost, carrying profound implications for endangered species conservation and marine biosecurity management.

The clinical presentation and local tissue reactions observed in *B. taipingensis* conform to the classical manifestations of "open gill disease" traditionally documented in freshwater aquaculture, including severe opercular flaring, respiratory distress, profuse mucus production, and extensive branchial chondrocyte remodeling (Khallaf et al., 2025; Mitchell et al., 2002; Van et al., 2012). This phenotypic uniformity indicates that the localized pathobiological cascade triggered by *C. formosanus* metacercariae is highly conserved across divergent teleost lineages. However, a major finding in the present study was the formation of prominent host-derived granulomas encapsulating individual metacercariae, accompanied by extensive tissue fibrosis and chronic inflammatory responses surrounding the cartilaginous support. Although Khallaf et al. (2025) noted similar phenomenon in wild tilapias, the mechanistic driver behind such extensive, non-localized tissue remodeling remains poorly understood. We hypothesize two distinct pathways responsible for this phenomenon: first, the metacercariae may secrete highly diffusible bioactive molecules, such as specialized proteolytic enzymes or immunomodulatory factors, which permeate through the interconnecting lacunae of the branchial extracellular matrix during active migration and early encystment (Khallaf et al., 2025). Second, as an evolutionary novel host encountering a heterophyid fluke in a brackish/marine environment, *B. taipingensis* may mount an uncontrolled, hypersensitive local immune cascade. This overactivated inflammatory response and granulomatous reaction likely extend far beyond the physical boundaries of the parasite capsule, leading to extensive lamellar atrophy and fusion, alongside severe mechanical occlusion and localized swelling of the gill apparatus, which represents the direct physiological cause of death via acute hypoxic suffocation (Lai et al., 2025; Thoney and Burreson, 1988).

Crucially, the standardized parasite burden recorded in *B. taipingensis* (3832 ± 473 PPF) represents one of the highest quantitative infection levels recorded among teleost fishes of comparable body size. To contextualize this magnitude, long-term epidemiological data from susceptible bighead carp (*Aristichthys nobilis*) demonstrated a peak mean infection intensity of 1878 PPF (Zeng and Liao, 2001), whereas surveys of wild Brazilian cichlids (*Australoheros facetus*) revealed a substantially lower mean intensity of 134 PPF (Pinto and de Melo, 2012). Strikingly, the parasite intensity harbored by *B. taipingensis* exceeds that of *A. facetus* by nearly 29-fold and doubles the peak load reported in cultured bighead carp, highlighting its exceptionally high susceptibility. Furthermore, established freshwater hosts exhibit a well-defined, size-dependent immune clearance mechanism against *C. formosanus*. In bighead carp, the parasite intensity drops precipitously once the body length exceeds 90 mm, and the branchial apparatus becomes entirely free of encysted metacercariae when the fish surpasses 140 mm (Zeng and Liao, 2001), a physiological clearance pattern mirrored in grass carp (Zeng and Liao, 2000). Paradoxically, *B. taipingensis* juveniles maintained an extraordinary parasite burden and suffered lethal consequences at a significantly larger size class (∼25 cm).

We propose two potential immunological hypotheses to explain this profound vulnerability and lack of growth-dependent clearance. First, *B. taipingensis* may possess an inherent defect in humoral immunity against branchial helminths. Early comparative studies on another marine sciaenid, the spot (*Leiostomus xanthurus*), demonstrated a complete failure to produce specific antibodies (as measured by ELISA) following prolonged exposure to gill parasites, alongside a total absence of localized leukocyte reactions at attachment sites, suggesting a family-wide constraint in antigen recognition or processing within sciaenid mucosal tissues (Thoney & Burreson, 1888). The encystment of *C. formosanus* within the deep primary cartilage may further sequester the parasite from systemic immune surveillance, preventing effective antibody-mediated clearance. Second, the capacity of *B. taipingensis* to establish robust, long-term immunological memory may be severely constrained. Recent functional characterization of adaptive immunity in a related sciaenid, the large yellow croaker (*Larimichthys crocea*), demonstrated that acute protozoan infection (*Cryptocaryon irritans*) induced rapid activation and proliferation of CD8^+^ T-cells, which was immediately followed by an irreversible cellular exhaustion and depletion phase within the spleen and head kidney, completely crippling long-term protective immunity (Lai et al., 2025). As a member of the same family, *B. taipingensis* may suffer from a similar, rapid exhaustion of its CD8^+^ T-cell pool upon exposure to heavy trematode antigens, precluding the development of growth-related resistance and resulting in chronic, high-load lethal accumulation.

The high cumulative mortality observed in this study aligns with historical global outbreaks where *C. formosanus* acted as a primary pathogen. In Brazil, intensive farming systems of ornamental platies (*Xiphophorus maculatus*) experienced sudden outbreak of *C. formosanus* with cumulative mortalities reaching 95% due to severe heterophyid encystment (Leibowitz et al., 2019). Similarly, a lethal outbreak of this parasite was documented in gray tilapia fry (*Oreochromis niloticus*) in the dry Pacific region of Costa Rica, where infection intensities averaged 8144–8216 PPF (Arguedas Cortés et al., 2010). These comparative cases underscore that *C. formosanus* represents an emerging, formidable biosecurity threat at the land-sea interface. Recent epidemiological evidence confirms that its primary gastropod host, *M. tuberculata*, has actively expanded its ecological range into low-salinity aquaculture systems (Lodeiros et al., 2025). Our findings demonstrate that the intrinsic euryhalinity of a marine fish, when paired with continuous brackish-water aquaculture practices, can inadvertently dissolve the geographical and ecological isolation that historically separated strictly marine-derived teleosts from freshwater pathogens. It is worth noting that another heterophyid trematode, *Haplorchis pumilio*, also utilizes the same snail intermediate hosts (*Melanoides tuberculata*) as *C. formosanus* and has been reported co-infecting fish in the USA, with metacercariae encysted along fin insertions—a site not examined in the present study (Huston et al., 2014). Future investigations should therefore include examination of fin tissues to rule out co-infections with *H. pumilio* or other heterophyids. Given that *B. taipingensis* is a critically endangered species undergoing intensive ex situ conservation, this parasite represents a major, newly identified barrier to population restoration and stock enhancement programs.

## 5 Conclusion

In conclusion, this study provides the first definitive evidence of natural *C*. *formosanus* infection associated with mass mortality in the critically endangered Chinese bahaba (*B*. *taipingensis*). The exceptionally high infection intensity (3832 ± 473 PPF) induced catastrophic branchial remodeling—predominantly characterized by host granuloma encapsulation, pronounced tissue fibrosis, lamellar epithelial hypertrophy, widespread lamellar atrophy and fusion, and severe localized swelling of the gill filaments—leading to respiratory failure. This finding expands the known host range and ecological distribution of this heterophyid trematode into brackish/marine aquaculture systems and highlights that parasitic diseases represent an important but underappreciated challenge in the conservation biology of endangered teleosts. Given these findings, future research should urgently prioritize practical biosecurity measures to interrupt the parasite's life cycle at the aquaculture facility level. A critical first step is to determine which intermediate hosts, including *Melanoides tuberculata*, are present in the culture ponds and surrounding water sources; if present, their distribution and abundance should be assessed. Subsequent efforts should focus on developing effective control strategies (e.g., physical removal, chemical mollusciciding, or biological control) and implementing strict biosecurity protocols to prevent the introduction and reinfestation of intermediate hosts. Breaking the life cycle at the level of intermediate hosts represents the most direct and feasible approach to managing *C. formosanus* infection in this specific aquaculture setting.

## CRediT authorship contribution statement

**Zhen Wu:** Writing – original draft, Visualization, Validation, Software, Resources, Methodology, Investigation, Formal analysis. **Liwen Xu:** Validation, Software, Resources, Methodology, Investigation, Formal analysis, Conceptualization. **Lin Yan:** Writing – review & editing, Resources, Methodology, Investigation, Formal analysis, Data curation. **ChiHai Ji:** Writing – review & editing, Validation, Methodology, Investigation. **Peiqi Tang:** Methodology, Investigation, Data curation. **Juan Feng:** Writing – review & editing, Supervision, Conceptualization. **Kuoqiu Yan:** Supervision, Resources, Investigation. **Zhixun Guo:** Writing – review & editing, Supervision, Formal analysis, Conceptualization. **Yiqin Deng:** Writing – review & editing, Writing – original draft, Supervision, Project administration, Methodology, Investigation, Funding acquisition, Conceptualization.

## Ethics statement

All experimental protocols and methods in this study were approved by the Committee on Laboratory Animal Welfare and Ethics of South China Sea Fisheries Research Institute (nhdf2023-08).

## Data availability statement

The data that support the findings of this study are available from the corresponding author upon reasonable request.

## Declaration of competing interest

The authors declare the following financial interests/personal relationships which may be considered as potential competing interests:Yiqin Deng reports financial support was provided by the National Key Research and Development Program of China. Yiqin Deng reports financial support was provided by the Central Public-Interest Scientific Institution Basal Research Fund, CAFS. Yiqin Deng reports financial support was provided by the Innovative Team Building Project of Guangdong Modern Agricultural Industrial Technology System. Yiqin Deng reports financial support was provided by the Guangdong Basic and Applied Basic Research Foundation. If there are other authors, they declare that they have no known competing financial interests or personal relationships that could have appeared to influence the work reported in this paper.
