# Supplementary material for: First report of Centrocestus formosanus infection associated with mass mortality in the critically endangered Chinese Bahaba (Bahaba taipingensis)
Source: Int J Parasitol Parasites Wildl. 2026 Sep 1;31:101280. doi: 10.1016/j.ijppaw.2026.101280 (PMC13578469; doi:10.1016/j.ijppaw.2026.101280)
Supplement: Supplementary file 1 [file mmc1.docx]

**Supporting Information**

**Table S1.** Real-time quantitative PCR (qPCR) screening results for eight endemic marine fish pathogens across the spleen, kidney, and brain tissues of diseased *B. taipingensis*

| **Fish No.** | **Tissue** | **NNV** | **RSIV** | **CAPRV** | **CI** | **VH** | **MM** | **SI** | **NS** |
| --- | --- | --- | --- | --- | --- | --- | --- | --- | --- |
| 1 | spleen | - | - | - | - | - | - | - | - |
| 1 | kidney | - | - | - | - | - | - | - | - |
| 1 | brain | - | - | - | - | - | - | - | - |
| 2 | spleen | - | - | - | - | - | - | - | - |
| 2 | kidney | - | - | - | - | - | - | - | - |
| 2 | brain | - | - | - | - | - | - | - | - |
| 3 | spleen | - | - | - | - | - | - | - | - |
| 3 | kidney | - | - | - | - | - | - | - | - |
| 3 | brain | - | - | - | - | - | - | - | - |
| 4 | spleen | - | - | - | - | - | - | - | - |
| 4 | kidney | - | - | - | - | - | - | - | - |
| 4 | brain | - | - | - | - | - | - | - | - |
| 5 | spleen | - | - | - | - | - | - | - | - |
| 5 | kidney | - | - | - | - | - | - | - | - |
| 5 | brain | - | - | - | - | - | - | - | - |
| 6 | spleen | - | - | - | - | - | - | - | - |
| 6 | kidney | - | - | - | - | - | - | - | - |
| 6 | brain | - | - | - | - | - | - | - | - |
| 7 | spleen | - | - | - | - | - | - | - | - |
| 7 | kidney | - | - | - | - | - | - | - | - |
| 7 | brain | - | - | - | - | - | - | - | - |
| 8 | spleen | - | - | - | - | - | - | - | - |
| 8 | kidney | - | - | - | - | - | - | - | - |
| 8 | brain | - | - | - | - | - | - | - | - |
| 9 | spleen | - | - | - | - | - | - | - | - |
| 9 | kidney | - | - | - | - | - | - | - | - |
| 9 | brain | - | - | - | - | - | - | - | - |
| 10 | spleen | - | - | - | - | - | - | - | - |
| 10 | kidney | - | - | - | - | - | - | - | - |
| 10 | brain | - | - | - | - | - | - | - | - |

**Note:** -, negative; NNV, Nervous necrosis virus; RSIV, Red sea bream iridovirus; CAPRV, Carpione rhabdovirus; CI, *Cryptocaryon irritans*; VH, *Vibrio harveyi*; MM, *Mycobacterium marinum*; SI, *Streptococcus iniae*; NS, *Nocardia seriolae*.

**Fig. S1.** Histopathological assessment of visceral organs from diseased *B. taipingensis*. (a) Overview of liver tissue demonstrating well-structured, regular hepatic cords and distinct sinusoids, with no signs of vacuolar degeneration, focal necrosis, or inflammatory cell infiltration (20× objective, scale bar = 100 μm); (b) High-magnification view of the hepatic parenchyma confirming uncompromised cellular integrity(40× objective, scale bar = 50 μm); (c) Splenic parenchyma demonstrating normal red and white pulp distribution without nodule formation, macrophage aggregate proliferation, or necrotic foci (40× objective, scale bar = 50 μm); (d) Myocardium displaying regularly arranged, intact cardiac muscle fibers completely free of interstitial edema or leukocyte infiltration (20× objective, scale bar = 100 μm).
